# Dataset for distribution of INGI/RIME and SLACS CRE transposable elements in *Trypanosoma brucei* genome

**DOI:** 10.1016/j.dib.2015.10.040

**Published:** 2015-11-06

**Authors:** Mohd. Faheem Khan, Kush Shrivastava, Rebeka Sinha, Virender Kumar, A.K. Jaitly

**Affiliations:** a Department of Plant Sciences, M.J.P. Rohilkhand University, Bareilly, U.P., India; b Division of Animal Genetics, Indian Veterinary Research Institute, Izatnagar, Bareilly, U.P., India

**Keywords:** INGI/RIME, *Trypanosoma brucei*, TEs, SLACS CRE

## Abstract

The current dataset is generated via bio-computational approach by surveying of INGI/RIME and SLACS CRE transposable elements (TEs) in latest update of *Trypanosoma brucei* genome. The distribution dataset (Supplementary File 1) shows the chromosome wise distribution of INGI/RIME and SLACS CRE transposable elements with the status of their -5′ and -3′ ends, genomic coverage and further elemental description about the completeness on the element. The 5′ upstream flanking sequence of 100 bp was then analyzed to find out possible regions that could act as insertion hotspots. The Fig. 1 represents the ten different motifs found in the 5′ flanking region of the INGI/RIME and SLACS CRE elements. The Supplementary File 2 describes the distribution of these ten motifs in different locations in *Trypanosoma brucei* genome. These new locations where motifs were found may provide useful information to track the future transposition events of INGI/RIME and SLACS CRE elements in different Trypanosoma species.

**Specifications Table**

**Table:** 

Subject area	*Biology*
More specific subject area	*Bioinformatics*
Type of data	*Tables in MS Excel sheet and images in JPEG image*
How data was acquired	*Bioinformatics Approach: Searching of INGI*/*RIME and SLACS CRE through Local Alignment given by Smith and Waterman algorithm and thereafter Perl programming was used to extract and assemble information in MS EXCEL format*
Data format	*Filtered and analyzed*
Experimental factors	*Trypanosoma brucei genome obtain from NCBI*; *Sequences of INGI and SLACS were obtain from Repbase repository.*
Experimental features	*Genome-wide distribution profiling was done of INGI/RIME and SLACS CRE transposable elements which are interspersed in the Trypanosoma brucei genome. The task is performed by the bio module of Perl along with standalone BLAST. The classification of INGI and RIME elements* (*as given in “elemental segment” column of*supplementary file 1) *was done based on Bringaud et al.*[1]*. The elements are classified as RIME A, RIME B, complete INGI element* (*for approximately* 1–5250 bp) *and partial INGI elements* (*for elements having incomplete RIME A or incomplete RIME B or both incomplete portions*)*. The SLACS CRE elements having both complete ends were classified as complete and those having truncated ends were classified as partial. The 5′ upstream flanking regions was analyzed for prediction of motifs using MEME server The genome wide search for these motifs was done using FIMO server to find out their number of occurrence* (Fig. 1)*. These motifs may act as transposition templates for these elements and it is also possible that it may be involved in the future transposition events of INGI/RIME and SLACS CRE elements.*
Data source location	*NA*
Data accessibility	*The Genome of T. brucei were taken from NCBI repository*
***http://www.ncbi.nlm.nih.gov/Ftp*** (*Genome sequences*)
*INGI AND SLACS CRE Elements were obtain from Repbase*
***http://www.girinst.org/repbase*** (*INGI, SLACS CRE*)
*Motifs or Patterns mined through MEME server*
***www.meme.nbcr.net***
*Further pattern search in upstream nucleotide assembly of Trypanosoma brucei through FIMO Server*

**Value of the data**

1. The provided data represents the complete distribution of INGI/RIME and SLACS CRE transposable elements (TEs) in *T. brucei* genome (TREU927, strain 927/4GUTat10.1; last updated on 2014/08/07). INGI/RIME TEs showed variability in upstream pattern and were hence analyzed more exhaustively. These elements have reported to be inserted in variable surface glycoproteins regions (VSG genes) of different species of *Trypanosoma*. The proteins encoded via VSG are a key molecule for immune escape and parasitic success. Thus distribution and prediction of transposition events of INGI elements may play a role in explaining the VSG switching.
2. Transposable elements (TEs) are vibrant elements which are involved in reshaping of host genomes by generating different combinations and rearrangements with the capabilities to create mutation, disruption of genes, shuffling of existing genes and regulate their expression. Some parasites that infected different mammals have different families of transposable elements throughout evolution due to their contribution in gene composition and their regulation mechanism. The provided dataset will be very helpful for the researchers in these types of studies.
3. Ten significance motifs were also found in 5′ upstream flanking region of INGI/RIME and SLACS CRE elements. These motifs can be used as a hotspot for tracking of these TEs in *T. brucei* genome as well as in other species.

## 1 Data

Transposable elements have been a critical part of the eukaryotic genome perhaps since their very beginnings. Millions of years of evolution have given these elements a central role in the maintenance of chromosomes and genetic modulation. Here we surveyed and analyzed the distribution and insertion preferences of interspersed INGI/RIME and SLACS CRE TEs in *T. brucei*. We also discuss how these transposable elements select and identify the insertion sites by analyzing the 5′ upstream flanking regions of these TEs which may further reveal the architecture and evolutionary mechanism of *T. brucei* genome.

## 2 Experimental design, materials and methods

**Fig. 1:**
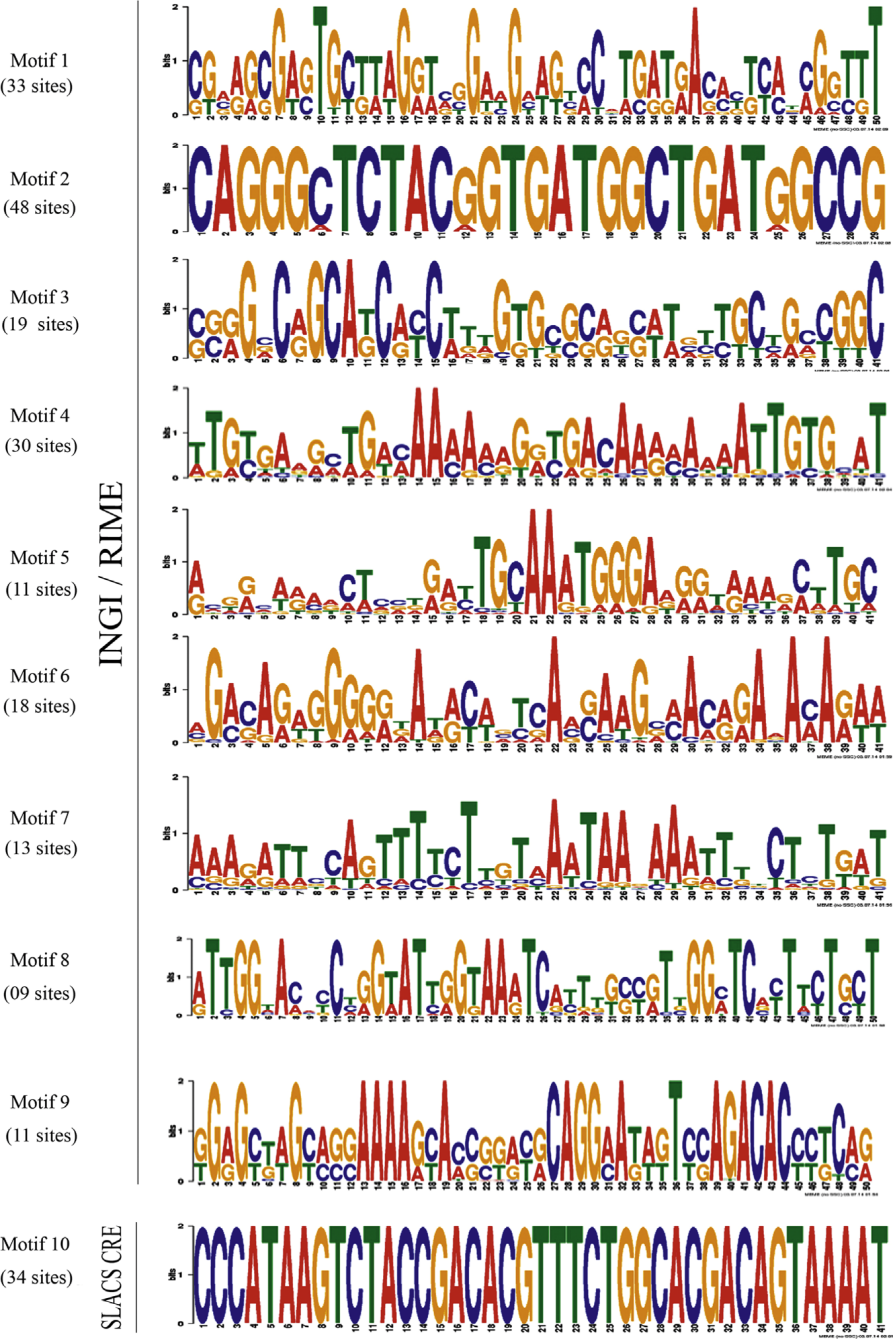
Identified motifs in 5′ upstream flanking region of INGI/RIME and SLACS CRE transposable elements. (Numbers in parenthesis represents the respective number of occurrence of each motif in the *T. brucei* genome).

The whole method of experimentation was completed in three segments. In the first segment, the copies of INGI/RIME and SLACS CRE transposable elements were tracked in the *Traypanosoma brucei* genome [2] via a standalone BLAST [3], [4]. Thereafter, the BLAST storage [5] was used to parse the report provided by the Standalone BLAST [4]. Significant information were extracted and assembled, these information provide the complete distribution of INGI/RIME and SLACS CRE under following captions like chromosome number, start and end position, their respective length, e-value and status of copies whether they are complete or abrupt and their further classification based on completeness of their ends. In the second segment, BioPerl scripts were developed for the extracting of 5′ upstream flanking region and status of INGI/RIME and SLACS CRE TEs [6]. In the third segment, the flanking sequences of INGI/RIME and SLACS CRE TEs were analyzed for searching of motifs by MEME server [7]. The results of MEME server were further used for searching of new locations of motifs in *Trypanosoma brucei* genome through FIMO (Find Individual Motif Occurrences) via Benjamini and Hochberg method [8], [9]. Details of all motifs are presented in Supplementary file 2, which have position-dependent scoring, score for each motif, locations of each motifs/pattern and its occurrence (Fig. 1 and Supplementary file 2) in *Trypanosoma brucei* genome.

## Figures and Tables

**Fig. S1:**
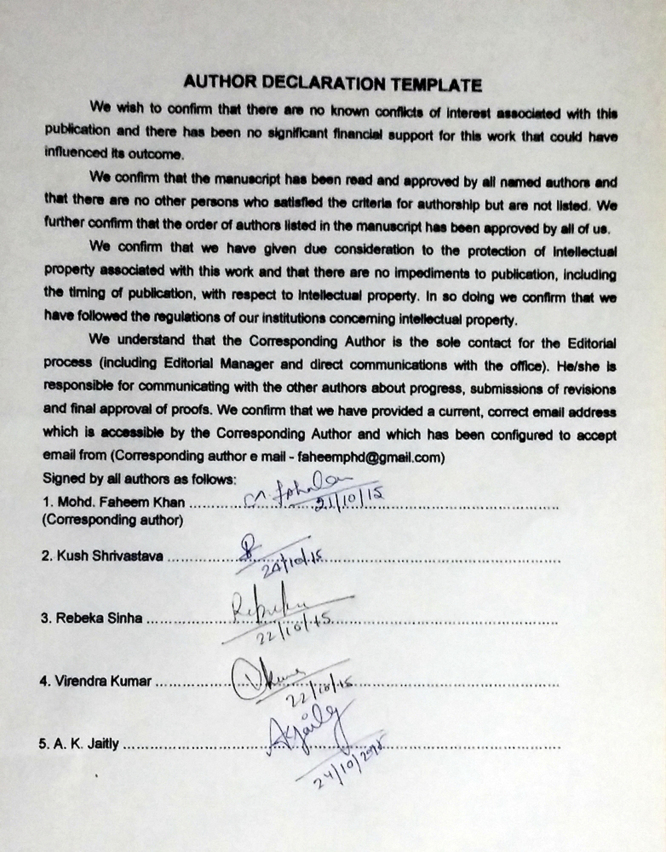


## Appendix A Supplementary material

Supplementary material Supplementary File 1: Distribution of INGI/RIME and SLACS CRE elements in *Trypanosoma brucei* genome.

Supplementary material Supplementary File 2: Genome wide distribution of ten identified motifs in *Trypanosoma brucei* genome.
